# Our New Volume

**Published:** 1853-10

**Authors:** 


### EDITORIAL DEPARTMENT.

*Our New Volume.*—We are entering upon a new volume, with new plans and new hopes. It is not to be denied, that the standard of eminence in dentistry has been gradually becoming higher and higher since the first foundation of this Journal. People demand of us more knowledge than they did twenty-five years ago, when a dentist stood but little higher in the social scale than a mere mechanical artizan. Without speculating on the causes of this advancement in the public demands, which has only gone on in the same ratio with the progress which dentistry itself has made, we are content to recognize and publicly acknowledge the fact.

In view of such a state of affairs, we have felt it to be our duty, as journalists, to take a step forward, in order to keep pace with this onward march of improvement. We have seen, as every thinking man must see, that quite a variety of information is necessary to the man who would successfully manage the teeth, whether he aims to preserve them from disease, or remedy the ill effects of morbid action, when that has once been set up. For this purpose he requires to understand all the modifications which these organs must suffer, from congenital vices of the system, or from acquired predisposition to disease, or from actual disease, or from functional derangement of the secretions, or from neglect of cleanliness and the consequent corrosive action of decaying food and putrefying secretions.

We presume that these premises will hardly be called in question, and if any of our readers should be disposed to doubt them, we ask him to sit down quietly and reflect upon the subject for twenty minutes; to consider the relations which these organs bear to the great function of digestion; how defectvie nutrition in early life must affect them; how manifold are their nervous sympathies which link them with so many and so remote organs, and, to come to their direct, plain and palpable connection with the secretions, how completely they are at the mercy of all acids which may be generated in the mouth, or rejected into it from the stomach.

Thus, without speaking of the many points in which anatomy, physiology and pathology touch the study of dental surgery, we see what a wide range of organic chemistry we require for the elucidation of the simple problem of the origin of caries. What agency does morbid saliva take in the production of this disease? How does decaying mucus act? What are the acids generated by putrefying food, and what is their action upon the teeth? How do these organs vary in different habits of body, and how are these varieties affected with the same acids? These are important practical questions, which must be answered by every man who is concerned about the prophylaxis of caries. And how wide a field of inquiry do they open! A very extensive investigation in the different departments of organic chemistry is required for this purpose. We must understand the chemistry of these solids and fluids, both in health and in disease, as the fundamental fact in our investigation. We must know what changes take place in the various articles of food, during their decomposition, and how they react upon healthy or diseased saliva. We therefore must have a very general acquaintance with organic chemistry, the office of which is to teach us these things.

But there is another class of inquiries equally important to the scientific dentist. How do these same acids, so generated, affect the materials introduced into the mouth to rectify the defects induced by disease? Still further, what are the reactions of the metals themselves during the process of preparing them for introduction into the mouth? How can a definite alloy be made, and what are the best properties for it? What is the best compound for making artificial teeth? How do the various substances react on one another at a high heat? What are the coloring matters, and how are they prepared? Thus it will be seen that, if the practical man in the different departments of dentistry would succeed, he must look into the various departments of chemistry far more closely than he would, at first sight, suppose.

It is in view of such facts as these that we have determined to add three distinct Summaries to our present volume. These are, 1st, a summary of the practical improvements in dentistry, whether mechanical or surgical; 2d, a summary of the progress of anatomy and physiology, so far as they throw any light upon matters connected with this art; 3d, a summary of the progress of chemistry. This last will include all new facts in the chemistry of digestion and of the secretions and solids of the mouth, new facts in the comparative chemistry of the teeth, new processes of metallurgy, of electrotyping, of porcelain manufacture, and all improvements in the arts which may elevate that profession to which our Journal is specially addressed.

The present number is but an earnest of what we intend to do. We ask the attention of the profession to it, confident that they will find in it much new matter, not easily obtained elsewhere, and very important to all who desire to be scientific dentists. We feel that we are furnishing a Journal which deserves the patronage of a liberal and enlightened profession, and we look for that support which we think we have a right to expect. Let our old subscribers show this new number to their friends, and interest themselves to advance its circulation. The more they extend our circulation, the more will they increase our facilities for serving them, and the more advantage will they, themselves, derive.

